# IL-17 Enhances Chemotaxis of Primary Human B Cells during Asthma

**DOI:** 10.1371/journal.pone.0114604

**Published:** 2014-12-10

**Authors:** Rabih Halwani, Roua Al-Kufaidy, Alejandro Vazquez-Tello, Mary Angeline Pureza, Ahmed S. BaHammam, Hamdan Al-Jahdali, Sami A. Alnassar, Qutayba Hamid, Saleh Al-Muhsen

**Affiliations:** 1 Prince Naif Center for Immunology Research, department of pediatrics, College of Medicine, King Saud University, Riyadh, Saudi Arabia; 2 Pulmonary Medicine Department, University Sleep Disorders Center, College of Medicine, King Saud University, Riyadh, Kingdom of Saudi Arabia; 3 Department of Medicine, Pulmonary Division-ICU, King Saud University for health sciences, Riyadh, Saudi Arabia; 4 Division of Thoracic Surgery, College of Medicine, King Saud University, Riyadh, Saudi Arabia; 5 Meakins-Christie Laboratories, McGill University, Montreal, Quebec, Canada; Université Libre de Bruxelles, Belgium.

## Abstract

IL-17 is a pro-inflammatory mediator that is believed to play a critical role in regulating tissue inflammation during asthma, COPD, as well as other inflammatory disorders. The level of expression of IL-17 has been shown to be upregulated in lung bronchial tissue of asthmatic patients. Several reports have provided further evidence that this cytokine could play a key role in enhancing the migration of inflammatory as well as structural cells of the bronchial lung tissue during asthma and COPD. B cell infiltration to sites of inflammation during inflammatory disorders such as bowel disease, asthma and COPD has been reported. Accordingly, in this study we hypothesized that IL-17 may exert a chemotactic effect on primary B cells during asthma. We observed that B cells from asthmatic patients expressed significantly higher levels of IL-17RA and IL-17RC, compared to those of healthy subjects. Using an in-vitro migration assay, B cells were shown to migrate towards both IL-17A and IL-17F. Interestingly, blocking IL-17A and IL-17F signaling using either anti-IL-17R antibodies or MAP kinase inhibitors prevented in vitro migration of B cell towards IL-17. These observations indicate a direct chemotactic effect of IL-17 cytokines on primary peripheral blood B cells with higher effect being on asthmatic B cells. These findings revealed a key role for IL-17 in enhancing the migration of B cells to the lung tissue during asthma or COPD.

## Introduction

Th-17 cells and their characteristic cytokines IL-17A, IL-17F, IL-21 and IL-22 play a beneficial role in the host-defense response against extracellular bacterial and fungal pathogens. However, they are also major harmful promoters in the pathogenesis of many chronic autoimmune and allergic disorders, including inflammatory bowel disease, rheumatoid arthritis, multiple sclerosis, systemic lupus erythematosus, and allergic asthma [1]–[3]. Thus, Th17-derived IL-17A and IL-17F are at the basis of many diseases, and their effects are complex and multiple; in particular, these cytokines can induce the release of various pro-inflammatory mediators including chemokines, cytokines, and metalloproteinases in many cell targets [4]–[8]. Inflammation of the airways in asthmatics is promoted by a number of cytokines, chemokines, prostaglandins and other mediators secreted by inflammatory cells (e.g., lymphocytes, granulocytes) and by structural cells (e.g., airway epithelial, smooth muscle cells). Yet, the pathophysiology of asthma differs considerably among patients; such differences, observed in the degree of severity of asthma symptoms, are believed to be determined by the predominant pro-inflammatory cytokine profile in the airways’ patients [9]–[11]. For instance, most patients with well controlled asthma symptoms typically exhibit a prevalent eosinophil infiltration in the airway tissues, along with detectable Th-2-derived cytokines (IL-2, IL-4, IL-5 and IL-13) [9], [12]. In contrast, asthmatics with refractory asthma symptoms present generally a significant infiltration of neutrophils in the airways, and detectable levels of Th-17-associated cytokines (IL-17A, IL-17F, IL-21) [4], [6], [10], [11], [13]; in these patients, a preferential infiltration of neutrophils over eosinophils is driven by IL-17-stimulated airway epithelial cells via p38 MAPK, and release the chemokine CXCL8 (IL-8) that promotes granulocyte recruitment, particularly neutrophils [5], [14]–[15]. In vitro experiments also support the possibility that IL-17 could, directly or indirectly, support the recruitment of IgE+ antibody-secreting B cells in the airways, by stimulating airway epithelial cells to produce CCL28 chemokine [8]. These observations are in agreement with those obtained from a mouse model, in which adoptively transferred subset of T cells expressing the inducible T-cell costimulator (ICOS) that is critical for the expansion of Th-17 cells, promoted a remarkable infiltration of both T and IgE-allergen specific B cells in lung tissues [16]–[17].

IL-17A and IL-17F cytokine signaling is mediated by specific receptors composed of IL-17RA and IL-17RC subunits, which are expressed on the cell surface of many cell types, including airway epithelial, airway smooth muscle and microvascular airway endothelial cells [14], [18]–[23]. Recent in vitro evidence suggested that IL-17A and IL-17F cytokines can also regulate airway smooth muscle (ASM) cell migration by an autocrine mechanism that involves the upregulation of growth-related oncogene (GRO) family of chemokines (GRO-a/CXCL1, GRO-b/CXCL2, GRO-g/CXCL3) [24]. Importantly, it was also shown in vitro that IL-17A, IL-17F and IL-22 cytokines could exert a direct chemotactic activity on airway smooth muscle (ASM) cells; hence, augmented ASM cell mass and tissue remodeling of the airways in severe asthma and COPD patients could be explained in part, by the infiltration of ASM cells elicited by Th-17-associated cytokines [23]. Also, among the adaptive immune cells, B lymphocytes express high levels of IL-17RA receptors, and therefore respond to Th-17-derived cytokine stimulations [25]. Hence, IL-17 modulates B cell activation and promotes its proliferation [7], [26]–[27]. Th-17 cytokines also stimulate Ig isotype switching by upregulating activation-induced cytidine deaminase (AICD) gene expression, and enhance the production of autoantibodies in a rheumatoid arthritis (RA) BXD2 mouse model [26], [28]. Importantly, IL-17 cytokines coordinate the timely sequestration of responder B cells within the follicular light zone of germinal centers (GC), via upregulation of the regulators of G-protein signals (RGS) Rgs13 and Rgs16, thus suppressing B cell’s chemotactic response to CXCL12 and CXCL13, and ensuring their interaction with follicular Th cells to promote B cell differentiation into autoantibody-producing plasma B cells in a RA BXD2 mice model [21], [28]. Similarly, the development of lung ectopic lymphoid tissues containing B cell follicles during infection (e.g., tuberculosis) or chronic inflammation (e.g., rheumatoid arthritis, COPD), is driven by CD4+ Th-17 cells and associated IL-17 cytokines, by promoting CXCL13 release and B and T cell infiltration [29]–[30]. Furthermore, in IL-17-deficient mice, production of antibodies is impaired, whereas IL-17RA-deficient mice are completely protected against collagen-induced arthritis, which highlights the importance of this cytokine pathway in B cell activation [31]–[32].

For many years, B cells were traditionally associated mostly with the production of antibodies. However, recent investigations have underlined their importance as modulators of the immune response in many chronic allergic and autoimmune diseases, through the release of cytokines and other inflammatory mediators. For example, B cells are important as a source of many regulatory cytokines such as TNF-alpha, lymphotoxin, IL-2, IL-6, IL-10, IL-13 and IL-17E among others [33]–[36]. Recently, we demonstrated that highly purified CD19+/CD20+ B cells express mRNA and proteins of IL-17A and F cytokines, which can be upregulated by TGF-beta, IL-23 and IL-6 cytokines [37]. In the current study, we propose the possible chemotactic role of IL-17 cytokines in regulating the infiltration of B cells in airway tissues. These findings will improve our understanding of the mechanisms by which IL-17 drives asthma pathogenesis, and may help in identifying critical molecules as potential therapeutic targets.

## Materials and Methods

### Subjects characteristics

Ten severe asthmatic patients (6 males and 4 females, mean age: 32.3±3.2 years) were recruited for this study. Refractory asthma classification was according to ATS criteria (ATS 1987) [38]. The criteria of recruitment were as follows: Severe asthmatics patients must take a high-dose inhaled corticosteroid: Budesonide 160 µg/twice a day (or equivalent), or daily anti-leukotriene for >6 months per year, and at least 1 other add-on therapy on daily basis for the previous 12 months. They were also required to fulfill at least two of the following criteria: daily use of a short-acting β-agonist; have persistent FEV1 <60% and FEV1/FVC <75% predicted; 1 urgent visit or at least 3 steroid bursts in the previous year; prompt deterioration with <25% steroid dose reduction, or previous near-fatal asthma within the last 3 years. Subject characteristics are summarized in table 1. Exclusion criteria included smoking history or any other pulmonary diseases or co-existing medical conditions such as cardiac and renal diseases and uncontrolled hypertension. Ten healthy control subjects were also recruited (6 males and 4 females, mean age: 33.4±3.7 years). All normal control subjects were non-smokers with normal lung function, no history or symptoms of allergy and respiratory diseases, and were not taking any medications for the preceding four weeks. The study protocol was approved by the Institutional Review Board (IRB) of the college of Medicine, King Saud University (Protocol number E-11-555). All patients and control subjects in this study signed an informed consent approved by the IRB.

**Table 1:** Demography and spirometry data of the recruited subjects.

	Asthmatics	Healthy Controls
Age (years)	32.3±3.2	33.4±3.7
Males/females	6/4	6/4
Atopy (% of total)	80%	0
Duration of disease (years)	14.3±7.6	n/a
FEV1 (mean±SD)	58.65±2.56	105.3±6.51
Medications	Inhaled corticosteroids: Symbicort II (Budesonide/Formoterol)(160/4.5 ug) (1–2 inhalations twice daily); Anti-leukotriene:Singulair (montelukast sodium) (10 mg/d); Ventolin (albuterol)(as needed).	No

Except for the medications indicated above, the patients were not receiving any other immunosuppressive drugs. *FEV_1_* forced expiratory volume; *FVC* forced vital capacity.

### Isolation of peripheral blood B cells

Peripheral venous blood (20 ml) was drawn from patients and control subjects. Blood was layered over Ficoll gradient (Axis shield, Norway) and centrifuged at 1000 g for 30 minutes. The mononuclear cells layer (PBMC) was then collected and B cells were isolated by negative selection using EasySep Human B cell enrichment kit (StemCell, cat #19054). B cells purity was consistently >98% as evaluated by FACS analysis and the viability of freshly isolated B cells was >99% as evaluated by trypan blue dye exclusion. All experiments were performed using B cells isolated from the same 10 asthmatics and 10 control subjects.

### RNA extraction and real-time RT-PCR

B cells were stimulated with cytokines Th17 (50 ng/ml)) for 12 hours prior to cell harvest. Cells were then harvested, total RNA extracted (amounts of total RNA extracted from 2×10^6^ B cells were 19.2±2.6 µg for asthmatic eosinophils and 15.9±2.1 µg for healthy controls) (RNeasy Mini kit, Qiagen, CA, USA). Levels of expression of IL-17RA and IL-17RC mRNA were then determined using quantitative RT-PCR (Applied Biosystems, 7900 Fast RT-PCR system). Relative expressions of IL-17RA and IL-17RC genes normalized with GAPDH were determined by the delta-delta Ct method [39].

### Flow cytometry assay

Isolated B cells (5×10^5^) were stimulated or not with Th-17 cytokines for 24 hrs and then stained for IL-17RA and IL-17RC: cells were incubated for 1 hour on ice with the primary antibodies (anti-IL-17RA, anti-IL-17RC, or corresponding Ig isotype controls) conjugated with phycoerythrin (R&D Systems). Cells were washed using 1xPBS and fixed in 2% paraformaldehyde and analyzed by flow cytometry (BD LSRII; BD Biosciences, Franklin Lakes, NJ). Results were analyzed using DIVA software (BD Biosciences) and are presented as a percentage of positive cells.

### Chemotaxis assays

B cells migration assays were performed as previously described [40] using a 48-well micro Chemotaxis Boyden Chamber (Neuro Probe, Inc, Cabin John, Md). Isolated B cells were growth-arrested in RPMI media supplemented with 0.1% FBS for 4 hours. A 5-µm diameter pore polycarbonate membrane (Neuro Probe) was treated with 0.01% Collagen type I (rat tail; Nalgene Culture 3D Matrix, Rochester, NY) solution in 0.01N HCl and placed between 2 chambers (8 mm^2^/chamber). B cells were added to the upper chamber (50×10^3^ cells), and different concentrations (1, 10, and 100 ng/ml) of cytokines (IL-17A and IL17F, alone or combined), or medium alone were added to the lower chamber. A preliminary experiment was performed to establish the time course of migration (4, 6, 18, and 24 hours). Because the maximal increase in migration was observed at 18 hours (data not shown), all subsequent experiments were performed for 18 hours. After 18 hours of incubation at 37°C, the membrane was removed, and its upper face was scraped clear of cells. Cells that migrated to the lower side of the membrane were fixed and stained with the Hema 3 kit (Fisher, Kalamazoo, Mich), according to the manufacturer’s instructions. The number of cells was counted in 5 random fields at a magnification x3200. Since B cells are non-adherent cells, migrating cells were also counted in the lower chamber by flow cytometry, and the total number of cells from both at the membrane and in lower chamber medium was obtained.

### Receptor activation

To confirm that the increased migration associated with administration of exogenous Th17 cytokines was dependent on receptor activation, we pretreated B cells with anti–IL-17RA or anti–IL17RC mAb (R&D Systems) prior to migration assay. Results were compared with the corresponding Ig isotype controls.

### Direct chemotaxis examination

To investigate whether the chemotactic effects of exogenous Th17 cytokines could be mediated indirectly by stimulating the release of chemokines from B cells, we added either mouse anti-human CXCL8 (IL-8) (10 µg/ml) or anti-human CXCL-13 (0.3 µg/ml) monoclonal antibodies (R&D Systems) into both the upper and lower chambers during the migration, to block the potential effects of IL-8 and CXCL-13. In each case, results were compared with the effects of adding the corresponding Ig isotype controls. In an alternative approach, asthmatic B cells were stimulated with IL-17A, F or A+F cytokines (50 ng/ml) for 18 hrs. Supernatants were then collected and incubated or not with anti-IL-17A, F, or A+F (R&D Systems) neutralizing antibodies for 2 hr. The ability of these supernatants to enhance the migration of asthmatic B cells was then tested using Boyden chamber.

### Signaling

To determine the pathways involved in IL-17 induced B cell migration, we used pharmacologic inhibitors directed against various pathways that are potentially known to be activated by IL-17 and may be involved in cells migration. B cells (50×10^3^) were incubated with the inhibitors for 1 hour at 37°C prior to migration assay. Specifically, we used the p38 MAPK inhibitor SB203580 (0.1 mM; Axon Medchem, Groningen, The Netherlands), the extracellular signal-regulated kinase (ERK) 1/2 MAPK inhibitor PD184352 (2 mM; United States Biological, Inc, Swampscott, Mass), the NF-kB inhibitor PS1145 (10 mM; Professor Sir Philip Cohen), and the phosphoinositide 3-kinase (PI3K) inhibitor PI103 (5 mM; Cayman Chemical, Ann Arbor, Mich). All results were compared with the corresponding vehicle control dimethyl sulfoxide.

### Assessment of p38 MAPK phosphorylation by western analysis

2×10^6^ B cells were starved using medium with 0.1% FBS for 18 hours. Cells were stimulated with 50 ng/ml IL-17A and IL-17F for 0, 10, and 20 minutes and total proteins were extracted using lysis buffer (1% Triton X-100 containing protease and phosphatase inhibitor cocktails (Roche, Mannheim, Germany). Protein lysates (10 µg) were then resolved on 10% acrylamide SDS-PAGE gel and blots were probed with antibodies to phosphorylated p38 MAPK (Millipore) and total p38 MAPK (Millipore). Membranes were analyzed with an Odyssey IR scanner using Odyssey imaging software 3.0 (LI-COR Biosciences, Inc).

### Statistical Analysis

Statistical analysis for cytokine induced migration was first performed within the subgroups by using ANOVA and, when significant, followed by the post hoc Bonferroni test. The paired t test was performed for all other assays. P value of <0.05 was considered statistically significant. All data are presented as means ± SD.

## Results

### IL-17 receptors are upregulated on asthmatic B cells

**Figure 1:**
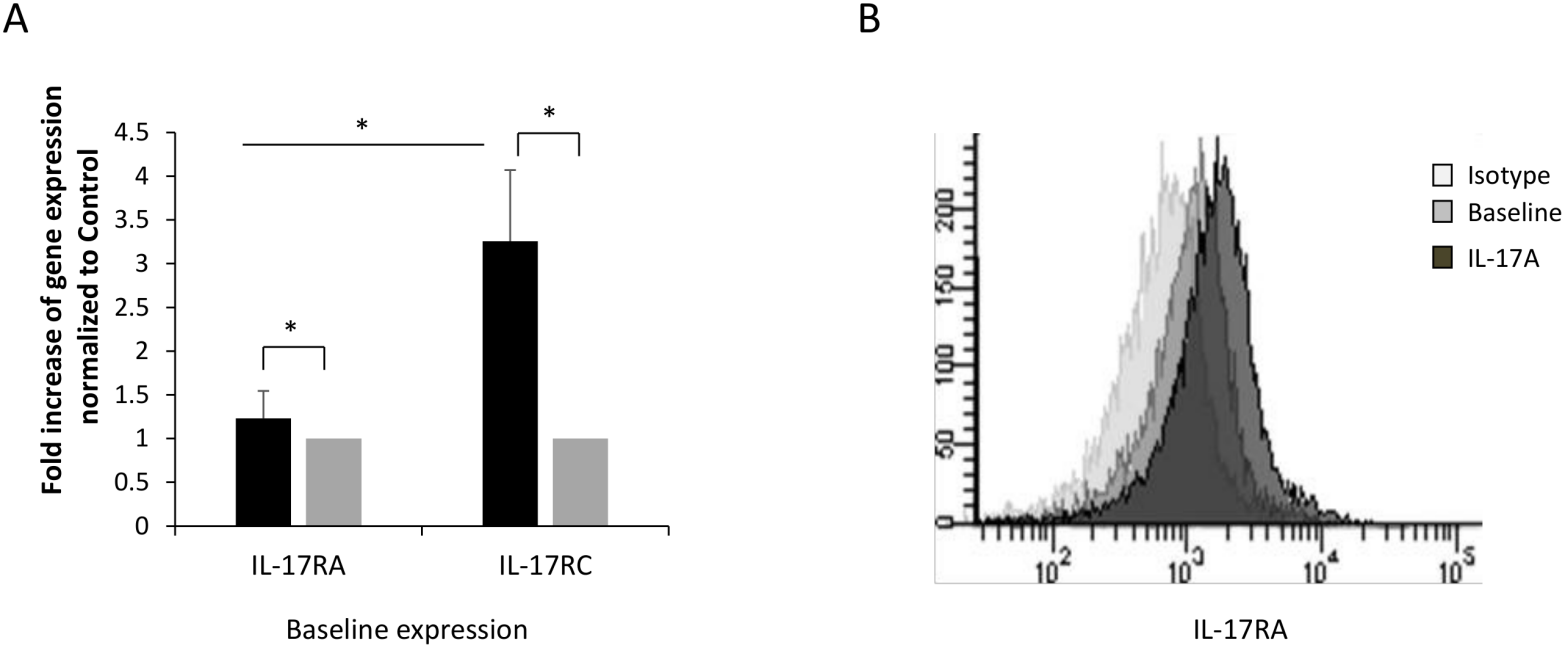
B cells express IL-17RA and IL-17RC. (A) Baseline mRNA expression levels of IL-17RA and IL-17RC on peripheral blood B cells isolated form asthmatic as well as healthy controls (n = 6). (B) representative histogram of IL-17RA expression on asthmatic B cells analyzed using flow cytometry. Data is presented as fold increase in gene expression normalized to control. Data is expressed as means ± SD. *P<0.05 compared to the control condition.

IL-17 cytokines were recently shown to be upregulated in airway tissue during asthma and to play critical roles in airway tissue remodeling. This includes enhancing the migration of structural and inflammatory cells such as smooth muscle cells, and neutrophils [14], [23]. To investigate the possible role of IL-17 in enhancing B cell migrations to bronchial tissue during asthma, we first isolated B cells from peripheral blood of asthmatic and healthy subjects and determined the level of expression of IL-17R on those cells using RT-PCR as well as flow cytometry. As shown in Fig. 1A, the baseline expression of IL-17RA and IL-17RC on B cells isolated from asthmatic individuals was significantly higher than that of healthy controls (IL-17RA: p = 0.04; IL-17RC: p = 0.005). In addition, IL-17RC was expressed at a significantly higher levels than IL-17RA (p = 0.003) on asthmatic B cells. Interestingly, stimulating peripheral blood B cells of asthmatic or healthy subjects with IL-17A or IL-17F or both resulted in a relative increase in IL-17RA and IL-17RC expression (data not shown). A representative histogram of IL-17RA expression on baseline and following stimulation with IL-17A is shown in Fig. 1B.

### Th-17 cytokines induce the migration of B cells in vitro

**Figure 2:**
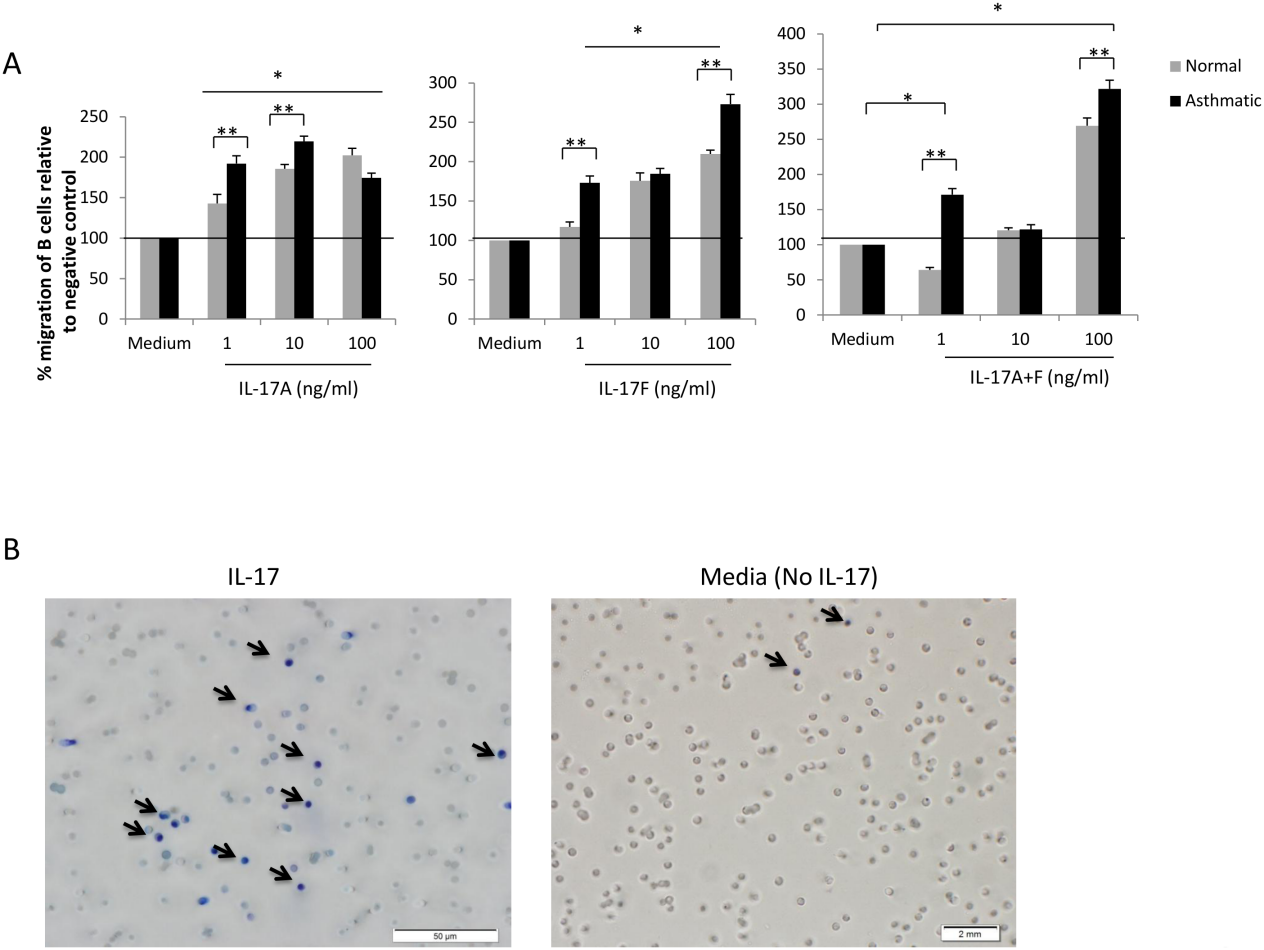
Th-17 cytokines enhance B cell migration in vitro. (A) Migration of asthmatic and healthy B cells towards a gradient of IL-17A, IL-17F, or IL-17A+F in vitro (n = 6). (B) Representative picture of B cells migrating towards IL-17A cytokine. Data is presented as % migration of B cells relative to negative control (medium). Data is expressed as means ± SD. *P<0.05 compared to the control condition (negative control). **p<0.05 comparing two groups.

We next examined the ability of IL-17 cytokines to directly induce the migration of B cells in vitro using Boyden chamber migration assay. As shown in Fig. 2A, IL-17A significantly induced migration of B cells isolated from asthmatic patients at a concentration as low as 1 ng/ml (192%±5.8, p<0.05). A higher chemotactic effect was observed at 10 ng/ml (219.6%±6.7, p<0.05), but not at 100 ng/ml (174.5±3.4, p<0.05). The chemotactic effect of IL-17A was also observed in B cells isolated from healthy subjects, with highest migration at 100 ng/ml (202.3%±2.5, p<0.05). IL-17 effect on B cells isolated from asthmatic patients was significantly higher than that on B cells isolated from healthy subjects at 1 and 10, but not 100 ng/ml concentrations. Similarly, IL-17F induced migration of B cells isolated from both asthmatic and healthy subjects, although this effect was significantly higher on B cells isolated from asthmatics; the lowest concentration (1 ng/ml) induced significantly the migration of asthmatic (173%±8.7, p<0.05) but not healthy B cells (117%±1.7, p = ns). Higher chemotactic impact of IL-17F on B cells was observed at 10 and 100 ng/ml (Fig. 2A). An additive effect was also observed when combining both IL-17A and IL-17F especially at 100 ng/ml (A: 321%±12.4, p<0.05; C: 269%±11.1, p<0.05). (Fig. 2A). A representative data of migrating B cells detected on the membrane facing IL-17A cytokine is shown in Fig. 2B.

### Signaling through IL-17 receptor is required for B cell migration

**Figure 3:**
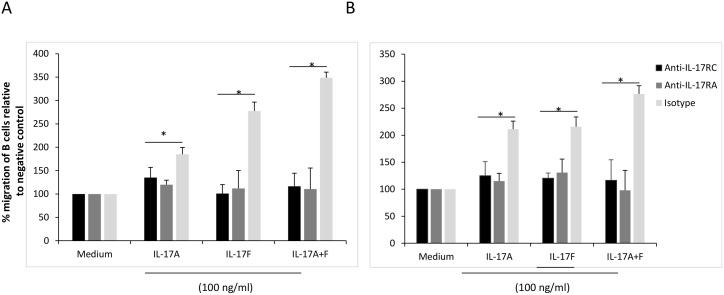
Signaling through IL-17R is required for B cell migration. B cells isolated from asthmatic (A) or healthy (B) subjects were treated with anti-IL-17RA or RC blocking antibodies or their isotype for 2 hrs prior to incubation with Th-17 cytokines for migration determination. Th-17 cytokines were used at 100 ng/ml. Data is presented as % migration of B cells relative to negative control (medium). (n = 7). Data is expressed as means ± SD. *P<0.05 is considered significant.

To confirm the requirement for activation of IL-17 pathway for B cell migration, we blocked IL-17R signaling on B cells using blocking anti-IL-17RA and anti-IL-17RC antibodies for 2 hrs prior to the chemotaxis assay with IL-17 cytokines. As shown in Fig. 3A, both IL-17A and IL-17F seem to signal through the two receptors and each receptor contributed to the increased migration of B cells. Blocking IL-17RA or RC signaling significantly inhibited asthmatic B cell migration induced by IL-17A (RA: p = 0.009; RC: p = 0.011), IL-17F (RA: p = 0.002; RC: p = 0.0005), or IL-17A+F (RA: p = 0.0002; RC: p = 0.0003), compared to Ig isotype control which had no effect on B cell migration. Similar results were obtained for B cells isolated from healthy subjects (Fig. 3B).

### Th17 associated cytokines induce B cell migration independent of IL-8, CXCL-13, or other possible B cell released mediators

**Figure 4:**
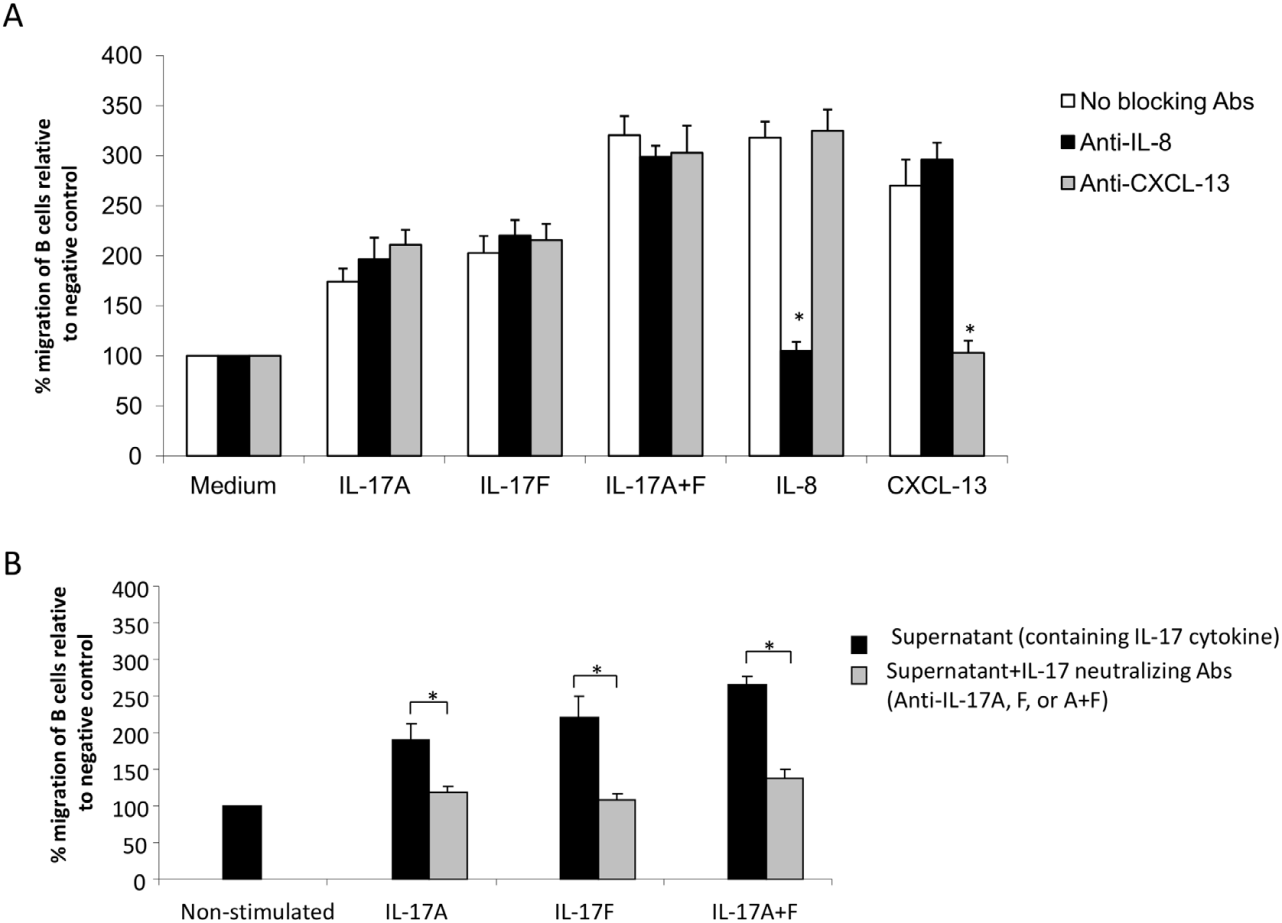
Th-17 cytokines induce a direct chemotactic effect on B cells. (A) B cells isolated from asthmatic patients were incubated with Th-17 cytokines (100 ng/ml), recombinant IL-8 (50 ng/ml), or CXCL-13 (50 ng/ml) in the presence, or absence, of anti-IL-8 (10 µg/ml) and anti-CXCL-13 (0.3 µg/ml) blocking antibodies in the medium of both upper and lower chambers of the migration assay. (n = 6). (B) Asthmatic B cells were stimulated with IL-17A, F or A+F cytokines (50 ng/ml) for 18 hrs. Supernatants were collected and incubated or not with corresponding neutralizing antibodies (anti-IL-17A, F, or A+F) for 2hr. Supernatants were then used in the lower chamber of migration assay against asthmatic B cells at the upper chamber (n = 6). Data is presented as % migration of B cells relative to negative control (medium). Data is expressed as means ± SD.

IL-17 could induce B cell migration indirectly by triggering the release of B cell chemoattractants from B cells. To investigate this possibility, we determined the migration of asthmatic B cells towards IL-17 in the presence of blocking antibodies to two known B cells chemoattractants, IL-8 and CXCL-13. As shown in Fig. 4A, anti-IL-8 or anti-CXCL-13 blocking antibodies did not prevent chemotaxis of B cells towards IL-17 cytokines although completely inhibited migration of B cells towards recombinant IL-8 and CXCL-13, respectively. Moreover, to rule out the role of other chemotactic mediators that could be released from B cells upon its incubation with IL-17, B cells were stimulated with IL-17A, F or A+F cytokines for 18 hrs; Supernatants were collected, incubated or not with anti-IL-17A, F, or A+F neutralizing antibodies for 2hr to neutralized the effect of exogenous IL-17 cytokines. The ability of these supernatants to enhance migration of B cells was then tested using Boyden chamber. As shown in Fig. 4B, exogenous IL-17 cytokines in the supernatant enhanced the migration of B cells (IL-17A (192%, p = 0.044), IL-17F (223%, p = 0.031), or IL-17A+F (268%, p = 0.017)). However, upon blocking the effect of exogenous IL-17 using neutralizing antibodies, supernatants of IL-17 stimulated B cells did not have significant chemotactic effect on asthmatic B cells (IL-17A (119%, p = NS), IL-17F (108%, p = NS), or IL-17A+F (138%, p = NS)) compared to baseline. This indicated that, upon exposure to IL-17 cytokines, B cells either did not secrete chemotactic mediators or secreted them at very low levels that may not contribute to the observed direct effect of IL-17 on B cell migration. This indicated that IL-17 cytokines induce the migration of B cells independently of mediators potentially released from B cells.

### Activation of p38 MAP kinase is required for Th17 cytokines induced B cell migration

**Figure 5:**
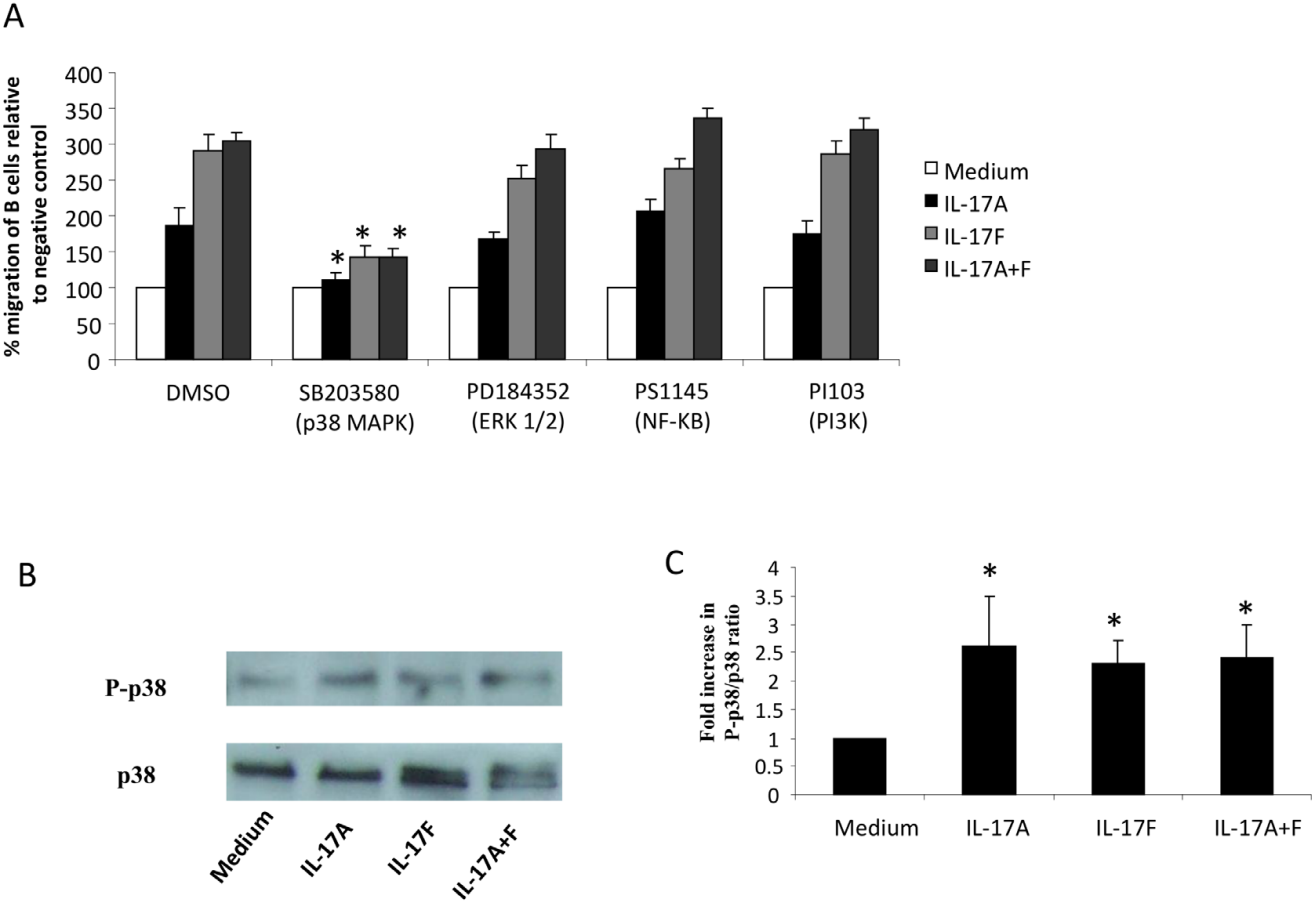
Activation of p38 MAP kinase is required for Th-17 cytokines migratory effect on B cells. (A) B cells isolated from asthmatic patients were pre-treated with different kinase inhibitors or DMSO carrier for 1 hr before being incubated with Th-17 cytokines in a migration assay. Data is presented as % migration of B cells relative to negative control (medium). (n = 6) (B) Western blots showing p38 MAPK phosphorylation in B cells following Th-17 cytokine stimulation. Asthmatic B cells (2×10^6^) stimulated with Th-17 cytokines were lysed using 1x RIPA buffer and cell lysates were resolved using western analysis. Blots were probed using anti-phospho-p38 and anti-p38 MAPK antibodies. (C) Ratio of p-p38 over p38 band intensity were determined using densitometer and data were presented as fold increase in p38 phosphorylation ratio following Th-17 cytokine stimulation compared to non-stimulated condition (medium). Data is expressed as means ± SD (n = 5). *P<0.05 is considered significant.

We next explored the signaling pathways involved in the Th17 cytokine–induced migration of B cells. Using pharmacologic inhibitors, we tested for the involvement of p38 MAPK, ERK1/2 MAPK, NF-kB, and PI3K pathways in the chemotactic effect of Th17 cytokines. An inhibitor of p38MAPK, SB203580, partially inhibited the migration of B cells induced by IL-17A (86.5%±9.7.0%, P<0.05), IL-17F (130%±6.9%, P<0.05) and IL-17A+F (142%±7.8%, P<0.05) (Fig. 5A). In contrast, inhibitors for ERK1/2 MAPK, NF-kB, and PI3K had no effect. For all Th17 cytokines, no inhibition was observed with either DMSO carrier or 100 ng/ml Pertussis toxin (data not shown). This result indicated that activation of p38 MAP kinase is required for Th17 cytokines-induced B cell migration. To confirm p38 activation, phosphorylation of p38 MAP kinase following Th17 cytokines stimulation was verified by Western blot analysis. As shown in Fig. 5B–C, stimulating B cells with 100 ng/ml IL-17A, IL-17F, or IL-17A+F cytokines for 15 minutes resulted in a significant increase in phosphorylation of p38 MAP kinase. P38 MAP kinase phosphorylation was also examined following 5, 10 and 30 minutes stimulation but highest phosphorylation levels were detected at 15 min. This data confirmed that Th17 cytokines induce B cell migration via the activation of p38 MAP kinase.

## Discussion

Ample evidence supports a major role for Th-17-derived IL-17A and IL-17F cytokines in promoting chemotaxis indirectly, via upregulation of a large number of chemokines, including CXCL8 (IL-8) and CXCL13, which are involved in neutrophil infiltration in sub-epithelial airway tissues of severe asthmatics, and T and B lymphocyte migration in follicular germinal centers, respectively [14], [24]–[25], [29]–[30]. Interestingly, a recent study suggested the intriguing possibility that IL-17 could exert a direct chemotactic effect on cells; reportedly, airway smooth muscle cells migrate in response to IL-17A and IL-17F gradient in vitro, and this mechanism was demonstrated to signal specifically through IL-17RA and IL-17RC receptors [23]. These findings open up new lines of investigation on the pathogenesis of severe asthma, which is characterized by neutrophilic infiltration and increased levels of Th-17-derived cytokines [6], [9]–[11]. In allergic asthma, B cell infiltration in sub-epithelial airway tissues has been considered mostly on the assumption that the main role is to produce antibodies [41]–[43]. However, accumulating evidence shows that infiltrating B cells in the lungs and other tissues could also be an important source of many cytokines, chemokines and other mediators of inflammation, and that their role as such has been long underestimated [35]–[37]. Therefore, our aim was to determine whether IL-17A and IL-17F cytokines directly induce chemotaxis in B cells from asthmatics and healthy controls.

This is the first report, to our knowledge, unambiguously indicating that Th17 cytokines directly exert chemotaxis of B cells in vitro. IL-17A and IL-17F led to enhanced migration of human B cells in a mechanism that involved direct signaling via IL-17R and activation of p38 MAP kinase. In the context of the growing recognition of the critical involvement of B cells in asthma pathogenesis, not only via the production of IgE, but of various regulatory mediators, our findings underscore the key role Th17-associated cytokines play in airway tissue remodeling and asthma pathogenesis.

Th17 cells are involved in a variety of human diseases, particularly in allergic and chronic autoimmune diseases [1]–[3], [44]. However, increasing evidences in the last few years demonstrated that these cells also contribute to asthma pathogenesis. For instance, the expression levels of IL-17 cytokines increase in lung tissue of asthmatics relative to disease severity [6], [9], [45]. In addition, Th-17 cytokines were shown to enhance the migration of neutrophils to the lung tissue [14] as well as to promote airway smooth muscle cells migration, proliferation and persistence [23]–[24]. They also activate Treg cells in asthma [46] and regulate the function of various structural and inflammatory cells involved in airway remodeling [13], [47].

In this study, we first confirmed the presence of Th17 cytokine receptors on B cells isolated from asthmatic and healthy recruited patients (Fig. 1A). Interestingly, IL-17RC is expressed on asthmatic B cells at a significantly higher level than IL-17RA and higher than IL-17RC on B cells from healthy controls. This could be due to the fact that IL-17RC expression is stimulated by IL-6, IL-23, as well as IL-17 cytokines all of which are upregulated in asthmatic tissue [54]–[55]. In addition, this finding explains the fact that migration of asthmatic B cells towards IL-17F is higher than that of B cells isolated from healthy controls especially at 100 ng where saturation could have been reached (Fig. 2A). The expression of both receptors was enhanced upon IL-17 cytokine stimulation. IL-17R was shown to be differentially expressed in various cells and tissues: IL-17RA is highly expressed in the bone marrow, thymus, and spleen but less easily detected in the colon, small intestine, and lung. Conversely, epithelial cells of the prostate, kidney, and joints express high levels of IL-17RC mRNA, while low levels of expression are detected in the hematopoietic cell compartments [48].

IL-17A and IL-17F cytokines promoted chemotaxis of B cells in vitro (Fig. 2A). These effects were reproducible and exhibited a dose-response relationship. Moreover, administration of neutralizing mAbs directed against both IL-17RA and IL-17RC abolished chemotaxis entirely, supporting the possibility that the chemotactic effect of Th17 cytokines is direct and specific (Fig. 3). However, IL-17A has been reported to release IL-8 from human endothelial cells [14] and ASMCs [15], [49]. IL-8 is known to be produced by B cells [50] and to induce its migration [51]. Similarly, CXCL-13 is a known chemoattractant of B cells and is produced by lymphoid follicles [29]–[30]. Given the potential for these chemokines to confound our results, we selectively used specific neutralizing antibodies to block IL-8 and CXCL-13 (Fig. 4A). We found no evidence to suggest that these chemokines account for our findings. Moreover, the supernatants of IL-17A/F-activated B cells (cleared from residual exogenous IL-17 cytokines) were not able to promote B cell migration (Fig. 4B) confirming the direct chemotactic role of IL-17 on B cells.

IL-17A and IL-17F homodimers have both been shown to bind independently to IL-17RA and IL-17RC [22]. However, whereas IL-17A and IL-17F bind IL-17RC with similar affinities, IL-17F binds IL-17RA with lower affinity [18]. As shown in Fig. 3, both IL-17A and IL-17F seem to signal through the two receptors and blocking any of the receptors dramatically inhibited the migration of B cells towards IL-17A or IL-17F. In fact, signaling through both receptors seems to be required for triggering B cell migration towards IL-17. However, since IL-17A and F bind with differential affinity to IL-17RA and IL-17RC, the presence of both cytokines seems to trigger the optimal signal required for maximal migratory capacity for B cells towards IL-17. Based on data reported in other systems, it has been argued that the binding of IL-17RC receptor might not be essential for the activity of IL-17A and IL- 17F [18]–[20], [48]. However, our results suggested a critical role for IL-17RC as blocking this receptor dramatically decreased B cells migration; and IL-17F induced higher migration of B cells even at low concentrations (Fig. 2A).

To explore the molecular mechanism underlying the chemotactic effect of Th17 cytokines on B cells, we tested the involvement of various MAP kinase pathways known to be activated by IL-17R triggering. Of all the MAPK inhibitors used, only SB203580, which inhibits p38α and p38β MAPK isoforms significantly inhibited B cell migration. Activation of p38 MAPK by IL-17A/F was further confirmed by the assessment of p38 MAPK phosphorylation. IL-17A and IL-17F exhibited similar kinetics for the activation of p38 MAPK, suggesting that they fulfill similar biological functions using the same signaling pathways. To our knowledge, this is the first demonstration of p38 MAPK activation by IL-17 in B cells, which is congruous with previous observations in microvascular airway endothelial cells, indicating that activation by IL-17 occurs in a p38 MAPK-dependent manner to upregulate endothelial adhesion markers (E-selectin, VCAM-1, and ICAM-1), and to improve IL-8 mRNA stability in smooth muscle cells [14], [15], [49]. In agreement with our observations and emphasizing the importance of p38 MAPK signaling, it was reported that chemotaxis of B cells in response to CXCL12, CXCL13 and CXCL21 is also dependent on the activation of p38 MAPK via the B-cell activating factor receptor (BAFF-R)/NF-kB pathway [52]. Interestingly, the levels of phosphorylated p38 MAP kinase, but not ERK or JNK, were shown to be correlated with B cells IgE production levels [53]. This may indicate for a role of IL-17 in enhancing IgE production within recruited B cells. Further investigations are required, however, to unravel this possible role of IL-17 cytokines.

In summary, we have demonstrated for the first time that IL-17A and IL-17F play a key role in inducing B cell migration. This novel observation, together with published evidence of chemotaxis activity by IL-17 on airway smooth muscle cells [23], suggests the possibility that other structural or innate/adaptive immune cells could display chemotactic response to Th-17-derived cytokines; if this hypothesis is true, the impact of Th-17 cytokines in asthma pathophysiology and in other chronic inflammatory diseases could have been underestimated. IL-17A and IL-17F exert their effects through signaling pathways that involve p38 MAP kinase activation. Our results provide a strong rationale for subsequent studies in animal models to assess the role of Th17 cytokines in B cell lung infiltration during asthma and COPD. These observations provide clear evidence for an important new mechanism for the promotion of lung inflammation and airway remodeling during asthma.
